# Experiences of participants in the co-design of a community-based health service for people with high healthcare service use

**DOI:** 10.1186/s12913-024-10788-5

**Published:** 2024-03-14

**Authors:** Deirdre McGowan, Claire Morley, Emily Hansen, Kelly Shaw, Tania Winzenberg

**Affiliations:** 1 Menzies Institute for Medical Research University of Tasmania, Hobart, Australia; 2 University of Tasmania, Hobart, Australia; 3 KP Health, Hobart, Australia; 4 Primary Health Tasmania, Hobart, Australia

**Keywords:** Co-design, High healthcare service utilisation, Health services, Qualitative research, Patient-centred care

## Abstract

**Background:**

Incorporating perspectives of health consumers, healthcare workers, policy makers and stakeholders through co-design is essential to design services that are fit for purpose. However, the experiences of co-design participants are poorly understood. The aim of this study is to explore the experiences and perceptions of people involved in the co-design of a new service for people with high healthcare service utilisation.

**Methods:**

A methodology informed by the principles of grounded theory was used in this qualitative study to evaluate the experiences and perceptions of co-design participants. Participants were healthcare professionals, health managers and leaders and health consumers involved in the co-design of the new service in Tasmania, Australia. Semi-structured interviews were conducted, and data were iteratively and concurrently collected and analysed using constant comparative analysis. Audio/audio-visual recordings of interviews were transcribed verbatim. Transcripts, memos, and an audit trail were coded for experiences and perspectives of participants.

**Results:**

There were thirteen participants (5 health professionals, 6 health managers and leaders, and 2 health consumers). Codes were collapsed into six sub-themes and six themes. Themes were bureaucracy hinders co-design, importance of consumers and diversity, importance of a common purpose, relationships are integral, participants expectations inform their co-design experience and learning from co-design.

**Conclusion:**

Most participants reported positive aspects such as having a common purpose, valuing relationships, and having a personal motivation for participating in co-design. However, there were factors which hindered the adaptation of co-design principles and the co-design process. Our research highlights that bureaucracy can hinder co-design, that including people with lived experience is essential and the need to consider various types of diversity when assembling co-design teams. Future co-design projects could use these findings to improve the co-design experience for participants, and ultimately the outcome for communities.

**Supplementary Information:**

The online version contains supplementary material available at 10.1186/s12913-024-10788-5.

## Background

A small portion of the population have disproportionately high levels of healthcare use and expenditure [1], termed high health service utilisation (HSU). This pattern is evident in health systems worldwide [2]. People who experience HSU are heterogenous in terms of demographic characteristics, and their mostly complex physical, medical, psychosocial, and spiritual care needs [3, 4]. Factors identified as contributing to HSU include unmet psychosocial needs, low socioeconomic status, limited access to primary and preventative health care, and distrust in the healthcare system [3, 5–7]. HSU detracts from optimal health system performance with overuse of valuable resources and is associated with poor health outcomes [8, 9]. However, the effectiveness of interventions to reduce HSU is limited [2, 10–13]. One reason for this may be failure to target and tailor interventions to the highly variable needs of people with HSU [2]. Thus, when (re)designing interventions for these people it is imperative to incorporate the diverse perspectives of end-users to ensure interventions are acceptable, feasible, and sustainable [5, 14].

Co-design is a methodological approach well suited to achieving this aim. The terms co-design and participatory design are often used interchangeably, however, while there are similarities there are important differences [15]. Co-design is a process of “*bringing consumers, carers, families, and health workers together to improve services. It creates an equal and reciprocal relationship between all stakeholders, enabling them to design and deliver services in partnership with each other*” [14]. Whereas, in participatory design end-users are involved as advisors only during the design process, and do not co-lead service (re)design, implementation, or evaluation [15, 16]. Co-design is increasingly popular in healthcare quality improvement and service (re)design [17, 18], however the ambiguous, broad nature of co-design has led to a multitude of definitions, variance in meaningful engagement, and use of ‘co- ‘ terminology in the absence of involvement of people with lived experience [15, 16, 19–21]. Examining the experiences of co-design participants is essential to understand if the co-design process embodies the desired principles of equal partnership, openness, respect, empathy and design together [14]. Despite this, few studies have formally evaluated participants experience of active involvement in the co-design of new services [8, 9], in primary care [8] or outside the United Kingdom and North America [22, 23].

To address these evidence gaps, we undertook the co-design of a community-based model of care to address the needs of people with HSU, in the form of frequent hospital admissions at a regional public hospital in Tasmania. This evaluation aimed to explore the experiences and perceptions of people involved in the co-design process.

## Methods

This is a qualitative study exploring the experiences and perceptions of healthcare professionals, policy makers, administrators and consumer representatives who participated in the co-design of Healthcare Connect North. The study methodology used components of grounded theory as described by Chun Tie et al. [24] and qualitative methodological rigor as described by Tobin and Begley [25]. Grounded theory was chosen to guide the research as we wanted to use open ended interview questions where participants were at the centre of that question. We also wanted to let the data speak for itself rather than impose ideas on how to approach the data [24]. In grounded theory data is iteratively generated so findings are ‘grounded’ in the data rather than researchers imposing a fixed idea or past experience on the data [24, 26]. Grounded theory is systematic, yet dynamic and iterative and provides a rigorous framework for information gathering, coding, and analysis, which is both inductive (bottom-up) and deductive (top-down) [24, 26]. Deductive and inductive thinking can both be used in the 'constant comparative analysis' phase of grounded theory, which is a cyclic iterative process in which codes and themes are compared, collapsed, and new data and codes are compared with existing codes and themes [24]. Qualitative study procedures were guided by the Consolidated Criteria for Reporting Qualitative Research (COREQ) guidelines [27] (Supplement 1).

The study received ethical approval from the Human Research Ethics Committee of the University of Tasmania, Australia (H0026675). The all-female investigator team comprised of two registered nurses (DM, CM), one of whom is a PhD candidate. One sociologist (EH) with expertise in qualitative research, and two general practitioners (GPs) (TW, KS) with expertise in public and primary health, one of whom was the principal investigator with a specific interest in chronic disease management.

### Setting and participants

In Australia, the federal government funds non-government, not-for-profit organisations called Primary Health Networks to co-ordinate and commission primary healthcare services for their communities [28]. There are 31 Primary Health Networks in total [29]. These work in partnership with stakeholders, providers and service users to deliver outcomes based on person- and community-centred needs assessment, prioritisation, procurement, monitoring and evaluation [30]. Healthcare Connect North is one such service which was developed and implemented by the Tasmanian Primary Health Network, Primary Health Tasmania with funding from the Australian Federal Government. Primary Health Tasmania chose to use co-design because in the context of commissioning, co-design enables person-centred, locally adapted approaches that are accessible, efficient, effective and comprehensive [31]. The co-design was undertaken with a governance structure comprising of a Steering Group, Advisory Group and Working Group (Table 1). Co-design participants included healthcare consumers, healthcare professionals (GPs, registered nurses, allied health professionals) and staff in health services management and leadership roles. They came from Primary Health Tasmania, the Tasmanian Department of Health, the Tasmanian Health Service, the University of Tasmania, private practice and Health Consumers Tasmania. The co-design process consisted of online meetings of Steering, Advisory or Working Groups, out of session communication (email and one-on-one meetings) and two workshops which included case studies and small group activities (March and April 2021). Co-design occurred during the COVID-19 pandemic which affected the timing and types of co-design activities that were undertaken. At the time of this study, Healthcare Connect North was in the early stages of implementation.

**Table 1:** Governance of healthcare connect north co-design^a^

	*n*	Attributes	Aim
Steering Group	9	Key decision makers from Primary Health Tasmania, the Department of Health, the Tasmanian Health Service, Health Consumers Tasmania	To provide strategic advice and deliver project outputs
Advisory Group^b^	13	Expertise in health planning, general practice, primary care, nursing, healthcare service (re)design and health consumer representation from Primary Health Tasmania, Department of Health, Tasmanian Health Service, University of Tasmania, and Health Consumers Tasmania	Provide advice and guidance to the Steering Group to aid the development and implementation of the service
Working Group^b^	12		Provide detailed concept design of the service model

^a^Some co-design participants were members of more than one governance group, the total number of individuals involved was 26

^b^Advisory Group and Working group merged in August 2021, *n* = 15

People from any of the three governance groups involved in the co-design process who attended one or more meetings or workshops were invited to participate in the study (*n* = 26). Two GPs who were co-design participants but also investigators in this study were excluded. Participants were recruited via an email invitation sent on behalf of the research team by a Primary Health Tasmania delegate. Participants that did not respond to the email within two weeks were sent a follow up email and/or a phone call. Those who elected to take part in the study were sent participant information and consent forms.

### Data collection and analysis

Components of grounded theory used were constant comparative analysis, memoing, data collection, coding (initial and intermediate), theoretical sampling and theoretical sensitivity [24]. An open-ended interview guide (Supplement 2) was developed to explore key areas of the co-design process. As is common in qualitative research and essential to grounded theory, the interview guide was iterative, flexible, and adapted as data collection progressed to follow data leads and reflect emerging themes or responses from interviewees [24]. The first author (DM) undertook the semi-structured interviews. Participants were interviewed once either in person or online according to their preference. Interviews were recorded (audio-visual or audio), transcribed verbatim with embedded transcription functionswithin MS TEAMS, Zoom or MS Word, and checked for accuracy. Participants received a copy of their transcribed interview and were able to clarify, change or delete any comments. Journal entries were taken after each interview and when reflecting on the data, documenting the interviewer’s evolving thoughts, interview adaptions, potential biases, and emerging themes.

Transcripts and journal entries were analysed using NVivo Release 1.5.1 (940). Two investigators (DM, CM) iteratively read and coded transcripts, initially independently and then meeting regularly to discuss code names and themes with the aim of understanding different perspectives and gaining consensus. The full investigator team met at regular intervals to refine the analysis. A thorough audit trail was maintained to trace the comparison of data, themes, and relationships. Data that reflected more than one code were coded multiple times. Data saturation was assessed as achieved when no new codes or themes were identified from interviews.

## Results

Thirteen co-design participants, two of which were healthcare consumer representatives, were interviewed from June to September 2022 (Table 2). Reasons for non-participation were too busy (*n* = 4), no reason provided (*n* = 8) or unable to contact (*n* = 1). Interviews were conducted via MS TEAMS (*n* = 10), Zoom (*n* = 2), or telephone (*n* = 1), in participants’ workplaces (*n* = 10) or home (*n* = 3). Interviews lasted on average 28-min (range 15 to 51-min).

**Table 2:** Characteristics of study participants (*n* = 13)

Characteristic	*N* (%)
Age	
45–54 years	5 (39%)
55–64 years	6 (46%)
> = 65 years	2 (15%)
Gender	
Female	8 (62%)
Background	
Health professional or researcher (e.g., GP, nurse, allied health)	5 (39%)
Health manager or leader (e.g., policy maker, health services manager, executive leader)	6 (46%)
Health care consumer	2 (15%)
Governance group^a^	
Steering Group	4 (31%)
Advisory Group	4 (31%)
Working Group	6 (46%)

^a^Some participants were in multiple governance groups

Codes were condensed into six themes and six sub-themes (Table 3). Participants comments relevant to these are italicised below, and further examples are presented in Table 4. Codes are presented in bold when mentioned in the text below.

**Table 3:** Themes, sub-themes and codes

Themes	Sub themes	Codes
Bureaucracy hinders co-design		Bureaucracy
		Back channelling
		Decision making
Importance of lived experience and consumers in co-design	Perspectives of people with lived experience is essential	Experience of chronic disease
		Consumers are valuable
		Consumer input underdone
		Consumers were not representative of people
		We need to work better with consumers
		with HSU
	Importance of diversity	Hearing others’ experiences
		Diversity
		Lack of diversity
Importance of a common purpose in co-design		Need for change and wanting to do things differently
		Person centred
		Funding
		Innovation in primary healthcare
		Personal contributions
Relationships are integral to successful co-design	Relationships in co-design	Relationships
		Building partnerships and/or relationships
		Collaboration
	Challenges to relationships	Staff turnover
		Different agendas
		Conflict
		Communication
Participant expectations inform their co-design experience	Expectations	Different emotions experienced during co-design
		Personal beliefs
		Expectations of co-design
		Tokenism
	Pace of change	Pace of change
		Momentum
		Engagement over time
		COVID-19 pandemic
Learning from co-design		What participants learnt
		Need to evaluate to inform change

**Table 4:** Example quotes from interviews

Themes	Sub- themes	Codes	Example quotes
Bureaucracy hinders co-design		Bureaucracy	*“…the processes actually just move so slowly…the checks and balances just get in the way sometimes, as far as it has to go up a pathway, there has to be a legal opinion, there has to be an accounting opinion and their structures just don’t promote innovation.”* Participant 1 *“To start off with everyone was really, really motivated. Everyone was really, really positive. Everyone was really keen to get it up and running. And then everything went quiet … and then bureaucracy took over.”* Participant 2 *“you’re dealing with big systems with very, very insular and entrenched processes. I mean dealing with [name of department]. Oh my God, that is just frustration personified. [Department] is so so slow to do anything… it’s just such a slow and antiquated process, but that’s what big systems are. They are that, they are their stable points. So unfortunately, you’ve just gotta bite your tongue and just grind through.”* Participant 7
		Back channelling	*“I think there were lots of discussions that happened outside of the group, sort of back channellings…A lot of backchanneling and a agreeing this is what it’s gonna be like, you know, this is what the service is gonna look like.”* Participant 10 “*Having more informal communication about things that I could potentially have helped with.”* Participant 5
		Decision making	*“The decisions I think were made by the core team. And I can only say that because we provided ingredients, and they came back with the cake. And I can’t even remember even where there’s an opportunity to sort of ask whether if it’s gluten free or had anything particularly in it. It was sort of, here’s a solution, or here’s what we’ve come up with.”* Participant 12 *“…sometimes I feel like it was tricky to get decisions until you got that CEO, PHN to CEO to Secretary…but then to get some of those things really actioned has required getting just a couple of key decision makers in the room who had that authority to go ‘Yes, we will do it.”* Participant 3 *“I did get the sense that there is a little bit of a preordained sort of approach to the workshop. That this had all been thought through already and we were sort of…being pushed down a pathway that had been sort of preordained…We’re not really designing the service or anything. It’s almost having a group of people in the room to validate the preordained process is the sense that I got.”* Participant 10 *“Decisions were generally, we would, as a project team we would propose and then people would discuss and request changes if they wanted changes. And then we would check in, do we agree? That would be how it was done.”* Participant 2
Importance of consumers and diversity	Perspectives of people with lived experience are essential	Consumers are valuable	*“There was some fantastic consumer input, I always enjoy that because they really get up, you know, they’re wise.”* Participant 4 *“Like…[GPs] have our perspective, your consumers have their perspective and they often, well it's always wise and good to listen to that perspective. So, I thought the consumers did a great job at coming up with ideas and saying this will work, that won’t work, have you thought of this. It was great.”* Participant 4 *“I think at one stage we had three or four different consumer reps and it was, it was wonderful to see that, you know, we weren't all sort of looking at the same thing. You know one would be talking about one part of the pie and then the other would be, you know, talking about their experiences and that would…be a different type of the cut up of pie of sorts and, so yeah, we weren't just sort of talking about the one thing.”* Participant 6 *“I think there is certainly a greater level of awareness and understanding from the consumer perspective and I think community and consumers are much…better prepared to accept different models of care now than they were say, 10 years ago. In fact, I think they are well ahead of where the provider community is.”* Participant 7
		Consumer input underdone	*"I feel like at times we're not sure how to do it [involve consumers] and so therefore we don't or we're just relying on you know, and don't get me wrong it's fantastic to have consumers as members of Advisory Groups…but it feels a bit at times, a bit one-dimensional.”* Participant 3 *“I think…there were a fair amount of assumptions made about and perhaps stereotyping of the people that we’re designing the service for.”* Participant 12 *“…it's a shame that we didn't actually get to more real consumers to be able to determine how the design might be better.”* Participant 2 *"The only area that maybe might have been a little bit underdone was that of community engagement and engagement with people who are suffering from chronic disease and what their experiences were and what they thought might be better to try and improve them.”* Participant 13 *“I don't know that we talked about lived experience much at all. I think it was more, it was focused on experiences with our services and service delivery and our clients.”* Participant 11 *“I don't think the model was road tested with consumers…ask them and talk to them and go through the process to see if it would work with them.”* Participant 12
		Consumer were not representative of people with HSU	*“I feel like it's a reflection of where we're at with the system, where it's not as comprehensive as it could be…I feel like at times we're not sure how to do it and so therefore we don't or we're just relying on you know, and don't get me wrong it's a fantastic to have consumers as members of Advisory Groups and, but it feels a bit at times a bit one-dimensional.”* Participant 3 *“I wouldn't say these consumers were poorly health literate, they were a bit more literate than the average probably.”* Participant 4 *“I mean high-flyers* [*people with HSU*] *aren't going to put their hand up and go pick me. That's just not the nature of the people I think”* Participant 12
		We need to work better with consumers	*“…we're still really learning about how to have consumers at the table as partners in planning and implementing and evaluating services. I think you know, we're making good steps, but you know it's not where it needs to be yet.”* Participant 3 *“…kitchen table sort of methodology, which is really just a focus group in an informal setting. And I think that would have been useful, and that's probably the best way to reach those, some of those consumers…I think having a parallel group you probably need more than one… so really like sounding boards or discussion groups where you can fly ideas with them to see how things might work … and then take that back to the group and unpack that about how the hospital and how the system deals with those issues that they raise.”* Participant 12 *“…focus groups or other mechanisms, I think would add value and we need we need to work out how we do it better.”* Participant 3 “…*induction and support…for health consumers”* Participant 12
	Importance of diversity	Hearing others’ experiences	*“I think you get a better understanding of how people get cared for outside of the system you work in”* Participant 1 *“…it's always interesting just to hear different perspective of the GP's and you know the different stakeholders in their management and interaction with client groups.”* Participant 11 *“I think it just made people sort of go, ‘Oh yeah’ and that was, you know, I think that was the point. Oh yeah, it's not quite like that, or yes, I was making assumptions.”* Participant 12
		Diversity	*“The different groups who were there…they also know different parts of the health system really well. And I think that helped to dissect some of the issues and pinpoint some of the problems that needed, big problems, that needed to be sorted out.”* Participant 12
		Lack of diversity	“*There's certainly diversity of background and skill set… outside white Anglo-Saxon males and females, there's probably not a great deal of cultural diversity there…”* Participant 7 *“Aboriginal and Torres Strait Islander, I can’t speak for them at all and I don't think we had anyone who's representing that group.”* Participant 4 *“I don't remember there being any sort of cultural diversity there of Indigenous, even gender [diversity] it wasn't really there at all.”* Participant 1 *“I think it was diverse amongst those people that actually were stakeholders in this process”* Participant 1 *“I call them the usual suspects and they're the people I see at all the committees” Participant 12* *“I think…there were a fair amount of assumptions made about and perhaps stereotyping of the people that we’re designing the service for.”* Participant 12
Importance of a common purpose		Need for change and wanting to do things differently	*“I think sustainability has been at the forefront of this one… I think that has meant people have thought more about sustainable patient focused care. And that's, I think for me that seems to be the thing that's had the greatest, hopefully the greatest impact on the outcome.”* Participant 12 "*putting old wine in new bottles”* Participant 4 *“…the actual co-design happened with the project team. Not back with us. So, we didn't, there wasn't this sort of ongoing cyclical relationship or iterative relationship with us in the co-design process. It was here, have your input and we'll go off and do something, then they came back with here’s the result. So, it’s, it had flavour of co-design about it, that's probably the best way to say it.”* Participant 12
		Person centred	*“And then we also had this opportunity of having a look at this de-identified data of a real person over a 12-month period and how many times they’ve been into the hospital and what their symptoms were and those sorts of things. And it really brought to bear that we, we were really talking about and trying to better somebody’s life. Whereas a lot of the time you do all of these governance documents or policy documents, and they're very high level and incredibly important, but an individual isn’t seen in those documents.”* Participant 6 *“…it's not a particularly sexy area. Do you know what I mean?…Like in hospitals, and some specialists that’s sexy, normally the surgeons and you know, the high fly dynamic one with Porsches and they cut people, so they're really important. Yeah, this group of people doesn't use that type of service. They're the non-sexy blocking up the wards type people and it's just nice that there's a bit of commitment and want to improve their journey, and we hope it will improve the experience for staff as well.”* Participant 2
		Funding	*“Literally, it's saying we've got $2,000,000. You can take it or leave it and we've got the resourcing that we want to apply for that…purpose…”* Participant 7
		Innovation in primary healthcare	*“I think we have got to a stage… in primary healthcare, where we can’t afford to not do something differently, we really got to that critical point and I think everyone's seeing that…we're all one health system and that, if you, if you don't actually engage with primary care and provide some different systems, then you can't solve the problems with emergency departments and beds.”* Participant 1 *“…very little opportunity for innovation in community care”* Participant 9 *“No, I didn't notice any different ways of working….the same group of people interacting in the same way, doing the same things. I didn't see anything different there.”* Participant 12
		Personal contributions	*“I think what I've bought is trying to remain focused on the difference we're trying to make”* Participant 3 *“Blood, sweat and tears…and pushing it, as in not letting it go… 'Cause at few points we could have just dropped the ball.”* Participant 2 *“Overall, I felt as though I didn't have a great deal of input to it because I didn't have a concrete understanding of what the final product was going to be.”* Participant 5 *"It was mostly the hospital people were talking.”* Participant 4
Relationships are integral	Relationships in co-design	Relationships	*“… it’s a bit about understanding the perspectives of the different stakeholders around the table and what’s in it for them.”* Participant 3 *“…you could feel it when you were talking to people. You could feel an underlying current.”* And *“’cause there’s a dynamic that we don’t necessarily know about…”* Participant 2 *“I think it is largely built on that notion of being able to trust”* Participant 7 *“…my impression is there’s been good debate and discussion, which is what you want…it’s not necessarily no news is good news, no news at times can be complete lack of engagement in the whole design process. So, I quite like seeing debate ‘cause I think that shows people are engaged.”* Participant 3
		Building partnerships and/or relationships	*“What we try to do is build partnership in the things that we can do that they can’t. So rather than saying ‘oh, we’re so different, we can’t work together’. We say, ‘we’re so different, isn’t that fantastic’. We can do stuff that you find too difficult, and you can get us to do stuff that you’re not able to do….”* Participant 7 *“…it’s about relationships and relationship building. And as frustrating sometimes as our respective systems might find each other… in Tasmania, it’s a lot about relationships. It’s who you know and how you engage with them to get them to trust the direction that you’re taking, but also to encourage them to maybe take risks or to do things that ordinarily they might not do if they didn’t know who they were dealing with. So, a lot of it is built on relationship”* Participant 7
		Collaboration	*"…who's paying for what and who's doing this and who's doing that…there seemed to be a bit of confusion around that, and it held up the project quite a lot.”* Participant 11 *“…my impression is there’s been good debate and discussion, which is what you want…it’s not necessarily no news is good news, no news at times can be complete lack of engagement in the whole design process. So, I quite like seeing debate ‘cause I think that shows people are engaged.”* Participant 3 *“Sometimes the pace with which we would like to work and the pace with which our partners can work are two very different things”* Participant 3
	Challenges to relationships	Staff turnover	*“I think the challenge has been there’s been quite a bit of turnover*…*it’s just that you get that really good traction and then the person changes and you sort of feel a little bit at the whim of that person, whether that’s still a priority or whether things have changed”* Participant 3 *“I remember one Advisory Group meeting and there was a…manager there and we'd finalised…[a] couple of the documents and they sort of said, ‘Oh no, can we go back over that?’…And you know, no we don't want to go back in time. You weren't at the other meetings, but your manager was…we're trying to move forward.”* Participant 9
		Different agendas	*“It had a very THS DHS [Department of Health] feel. So, it really, to my mind, is their agenda and the rest of us are sort of on the edges.”* Participant 4 *“…people were approaching it from their role and their experience, obviously. So, we have people who are on the ground or the consumers who were, I guess, checking what actually is feasible and what works and what people want. Whereas high level people are probably more looking at the system itself. But I think we were able to compliment the different skill sets and the different experiences. So, from my perspective it was a pretty beneficial process.”* Participant 8
		Conflict	*“I think there was potential for us to [have conflict] with [co-design participant]…so we made it our business to not have conflict and to clearly represent how we thought the services could complement each other rather than conflict each other. So, we anticipated a conflict and managed it through forming a relationship and clear communication.”* Participant 2 *“…there was potential for…it to turn into a research project rather than it being a service that's gonna, an operational service. And that was mentioned by people in the advisory group as well – this is a service, not a research project.”* Participant 2 *“I didn't notice any [conflict]. People are pretty grown up and polite.”* Participant 4
		Communication	*“…any other healthcare rep…I think would have had that difficulty in understanding the jargon, the process, the language and that sort of way in which people were talking to each other. I think they would have found that difficult.”* Participant 12 *“You know we communicated with them when it came to meetings, we communicated with them alright, but we didn’t, I don't think we ever offered more. So that whether or not people would have wanted more, I don't know. We didn't ask, ‘cause if you ask, they might want it and then you'll have to deliver it. And I probably didn't wanna know the answer.”* Participant 2
Participant expectations inform their co-design experience	Expectations	Different emotions	*“It was a mix of excitement and commitment and…not nervousness, but just wondering how it would go.”* Participant 3 *“I think we all got quite disappointed at times that things didn't look like they were going to happen.”* Participant 1 *“…there was certainly a high level of interest and a feeling of excitement”* Participant 9 *“An exercise in persistence and self-control.”* Participant 7
		Personal beliefs	*“That’s one thing I’m really keen to see…how do we demonstrate how we've made best use of all parts of the system.”* Participant 3 *“That was one of my things, is that how are we going to connect up those people who've got chronic disease management plans to, you know, having somebody come in and having an ACAT [Aged Care Assessment Team] analysis to see whether or not it's just that loneliness factor that is putting people back into hospital and those sorts of things.”* Participant 6 “*I have no idea [why I was asked to join].”* Participant 10
		Expectations of co-design	*“That we would start from scratch. That we would look at how all of these things, all of these organisations and committees that we've had in the past could link in those."* Participant 6 *“I was never really quite sure which, what role was expected of me.”* Participant 13 *“As I say, I thought it might be more about general practice, but it wasn't. So, whatever, it just went on its merry way.”* Participant 4
	Pace of change	Pace of change	*“…it's taking a long time. We, you know, when’s this rubber gonna hit the road.”* Participant 4 *“…let me say it's been long winded”* Participant 7
		Momentum	*“It's been a little bit stop start, I think that's largely COVID related.”* Participant 3
		Engagement over time	*“…a lot of enthusiasm to start with, a lot of enthusiasm at the moment, and I thought the process had died in the middle.”* Participant 1
		COVID-19	*“…unfortunately probably because of COVID, I was unable to give the level of engagement that probably would have been ideal…”* Participant 13 *“I feel like it's really hard to really estimate the level of influence COVID had on that, because we were, we'd, I think we'd started at pre-COVID and you know, so there was some challenges even before COVID started in terms of getting that, that momentum built.*” Participant 3 *“I mean in a different world, we probably would have had more face-to-face meetings and I think that would have, that would have made things a little bit better”* Participant 12
Learning from co-design		What participants learnt	*” I learnt a lot of things…from the data…it reinforced the social problems that people have that sometimes impact on their health care…I got to see a different side of the health system…how some of the different areas work and how they interact.”* Participant 8 *“I don't know that I, I mean you always learn something, but I couldn't, don't know what specifically.”* Participant 11 *“I think it's reinforced for me that system change is hard, but doable.“* Participant 3
		Need to evaluate to inform change	*“…I'm very much of the belief that we can keep adding services till the cows come home, if we don't look at how we are better joining up what we do and working on things together, then I feel like it doesn't matter how many services you put in, you're still gonna have big challenges. So…we can understand what works and what doesn't in terms of how we work together.”* Participant 3 *"…this is the trouble with government money, they give you money, but no real good way to do a program evaluation.”* Participant 4

### Bureaucracy hinders co-design

Most participants described some level of **bureaucracy** hindering co-design, such as frustration at the pace of change in large bureaucratic organisations, or hoops they had to jump through during the co-design process (Table 4).

*“…the processes actually just move so slowly…the checks and balances just get in the way sometimes…there has to be a legal opinion…an accounting opinion and their structures just don’t promote innovation.”* P1

The co-design governance structure was described as “*a fairly bureaucratic thing*” [P12]. Three participants described “***back-channelling***” [P10] as conversations or decisions occurring outside the co-design team. There was some confusion and dissatisfaction around **how decisions were made** (Table 4). Participants reported decisions were made by the core team, Steering Group or when the right person with decision making power was in the virtual room.

*“The decisions I think were made by the core team. And I can only say that because we provided ingredients, and they came back with the cake. And I can’t even remember even where there’s an opportunity to sort of ask whether if it’s gluten free or had anything particularly in it. It was sort of, here’s a solution, or here’s what we’ve come up with.”* P12

### Importance of lived experience and diversity

#### Perspectives of people with lived experience are essential

Most participants described personal or family experience of chronic disease, however professional participants described wearing their “*professional hat*” [P13] during the co-design process. Participants felt **consumers were valuable**, describing them as *“fantastic”, “wise”* [P4], and open to novel service delivery (Table 4). Most participants felt **consumer involvement was **“***underdone***” [P13] and expressed concern that **consumer representation was not as representative** of people with HSU as it could have been.

*“I feel like it's a reflection of where we're at with the system, where it's not as comprehensive as it could be…I feel like at times we're not sure how to do it…don't get me wrong it's fantastic to have consumers as members of Advisory Groups, but it feels at times a bit one-dimensional.”* P3

Three participants described how to **work better with consumers** in the future. They spoke about barriers of involving people with HSU, such as access to people with HSU, incentives, and health sector willingness to engage in consumer involvement.

*“…we're still really learning about how to have consumers at the table as partners in planning and implementing and evaluating services.”* P3

Others suggested using different mechanisms in the future to enable consumer involvement, such as kitchen table conversations, induction, and parallel reference groups (Table 4).

#### Importance of diversity

Many participants reported the value of **hearing others’ perspectives** and felt there was an adequately diverse mix of stakeholders and health professionals (Table 4). **Diversity** was most often described in terms of professional representation, but not in terms of other type of diversity (Table 4).

“*There's certainly diversity of background and skill set...outside white Anglo-Saxon males and females, there's probably not a great deal of cultural diversity there...”* P7

Participants noted **lack of diversity**, such as First Nations representation or people with mental health diagnosis (Table 4). Some participants felt the lack of diversity meant there were prescriptive ideas of models of care, or the service wouldn’t *“…hit the mark for the needs of the population”* [P5].

*“I think…there were a fair amount of assumptions made about and perhaps stereotyping of the people that we’re designing the service for.”* P12

### Importance of a common purpose

Most participants described a common purpose as **a need for change and wanting to do things differently** by bringing all sectors of the health system together to improve the experiences of people with HSU (Table 4).

*“I think we have got to a stage…in primary healthcare, where we can’t afford to not do something differently…I think everyone's seeing that…we're all one health system.”* P1

Participants considered workshops and case studies as instrumental in developing a **person-centred** common purpose.

*“We also had this opportunity of having a look at this de-identified data of a real person over a 12-month period…it really brought to bear that we were really talking about and trying to better somebody’s life.”* P6

**Funding** was viewed as a significant enabler for co-design. However, not all participants perceived **innovation**, describing the service as "*putting old wine in new bottles”* [P4] (Table 4). Most people felt they made a **personal contribution** (Table 4).

### Relationships are integral

#### Relationships in co-design

Participants viewed the building blocks of **relationships** such as trust, understanding and communication as integral to the success of co-design (Table 4). The co-design process was described by some participants as not just about current relationships, but about **building partnerships** and creating new or strengthening existing relationships.

*“What we try to do is build partnership…rather than saying ‘oh, we’re so different, we can’t work together’. We say, ‘we’re so different, isn’t that fantastic’. We can do stuff that you find too difficult, and you can get us to do stuff that you’re not able to do...”* P7

Participants spoke about the benefits and challenges of **collaboration**.

*"...who's paying for what and who's doing this and who's doing that…there seemed to be a bit of confusion around that, and it held up the project quite a lot.”* P11

#### Challenges to relationships

The challenge of **staff turnover** was raised by several participants. This had potential to put the program at risk if the co-design was not a priority for the new staff member(s), or new staff wanted to re-visit previously made decisions (Table 4).

*“I think the challenge has been there’s been quite a bit of turnover*…*it’s just that you get that really good traction and then the person changes and you sort of feel a little bit at the whim of that person, whether that’s still a priority or whether things have changed”* P3

Several participants spoke about **different agendas** people or sectors brought with them and understanding each other’s priorities (Table 4). The co-design team was described as “*grown up and polite*” [P4], there were only three examples of **conflict** raised in the interviews (Table 4). Several **communication** challenges were noted, such as intermittent communication, professional jargon, and acronyms.

*“…any other healthcare rep…would have had that difficulty in understanding the jargon, the process, the language and that sort of way in which people were talking to each other.”* P12

### Participants expectations inform their co-design experience

#### Expectations

Many participants had previous experience of co-design or service (re)design, described **different emotions** and a **personal belief** or motivation for participating in the co-design process (Table 4).

*“That's one thing I'm really keen to see…how do we demonstrate how we’ve made best use of all parts of the system.”* P3

Most participants described an **expectation** of working together from the start to design a person-centred community service. However, some participants described that they were “*never really quite sure what role was expected of* [*them*]” [P13] and some felt their inclusion in the co-design was **tokenistic**.

*“I sort of felt personally that you didn’t have a lot of agency in the process…it felt you’re just sort of there as a token figurehead.”* P10

#### Pace of change

All participants spoke about the slow **pace of change**, how long the process took and how this affected **momentum** and their **engagement over time** (Table 4).

*“…a lot of enthusiasm to start with, a lot of enthusiasm at the moment, and I thought the process had died in the middle.”* P1

Five participants spoke about the impacts the **COVID-19 pandemic** had on the co-design process, such as maintaining momentum, juggling involvement with managing the pandemic response, and interacting online (Table 4).

*“…unfortunately, probably because of COVID, I was unable to give the level of engagement that probably would have been ideal…”* P13

### Learning from co-design

Most, but not all participants felt they had **learnt **during co-design.

*” I learnt a lot of things…from the data…it reinforced the social problems that people have that sometimes impact on their health care...I got to see a different side of the health system…how some of the different areas work and how they interact.”* P8

Participants spoke about the **need to evaluate the co-design process to inform future co-design** initiatives. However, Government funding was perceived by one participant as a challenge to program evaluation (Table 4).

## Discussion

This study sought to explore the experiences and perspectives of people participating in the co-design of a community-based health service and contributes to the limited knowledge of the experiences and perspectives of co-design participants. Most participants reported positive aspects of their experiences such as having a common purpose, valuing relationships, and having a personal motivation for participating in co-design. However, they also identified areas that could potentially be improved. Bureaucracy, both internal and external to co-design was perceived to impact co-design. Participants valued the perspectives of consumers, however felt the perspectives of people with lived experience of HSU was missing. Participants felt that diversity in co-design team membership was important as it enabled participants to listen and understand diverse perspectives but described this co-design team as diverse in professional background only. Future co-design projects could use these findings to improve co-design experience for participants, and ultimately the outcome for communities.

Bureaucracy, either external to, or within the co-design process was described by many participants. Similar to other research [32, 33] participants described bureaucratic processes, such as checks and balances or calling on relationships to unblock stalled processes. Participants felt these bureaucratic structures could enable or hinder co-design and were inevitable in co-design across health sectors and with Government. This is consistent with previous co-design research [32, 33], which suggests implementing co-design methodologies into bureaucratic organisations poses challenges, as the culture and structure of bureaucratic organisations and co-design are completely different [33]. One participant described this co-design process as “a fairly bureaucratic thing” which may be the result of loss of “democracy” when moving co-design online during the COVID-19 pandemic. This highlights the potential need for blended delivery models of co-design which is fit for the post COVID-19 era [34, 35]. Participants described confusion over how decisions were made, such as needing to have the right decision-maker(s) in the room or decisions were made by the “core team”. Our findings suggest decisions were reliant on key individual(s) within organisations, which runs counter to co-design principles of design together and equal partnership [14]. This is consistent with other research, which suggests the locus of control or ultimate decision-making power often lies outside the control of co-design team members [16, 32] and risks the design process being participatory design rather than true co-design [15, 16]. This suggests co-design principles may not be able to be fully adopted in bureaucratic organisations, although research into how bureaucratic structures hinder and/or enable co-design is sparse [32]. As it is difficult to avoid bureaucracy when co-designing in healthcare or with Government, we recommend research to identify if co-design principles can be fully adopted in these structures and to understand how co-design teams navigate these complex environments [20, 32, 33].

Perspectives of people with lived experience of the issue at the heart of a healthcare service are essential in co-design, as their involvement can have powerful impacts on culture and shared understanding [14, 19, 36]. Participants used words such as *“wise”* and *“fantastic”* to describe consumer involvement and gave them (participants) an understanding of how people are cared for in the healthcare system. This is consistent with other research which found co-design participants became accountable to consumers, reduced conflict, and bought the human element to service (re)design [32, 33]. Yet, most participants in this study felt consumer involvement was “underdone”, “one dimensional” and not as representative of people with HSU as it could have been. This has been found in other co-design research which suggests perspectives of people with lived experience can be missing [37–39] and level of involvement can vary [19–21]. Consideration and communication of co-design definition, principles and methods is essential to ensure people with lived experience are not advisors, but co-lead design [19–21]. Failure to do so may unintentionally disenfranchise people with lived experience [38]. Several participants spoke about the challenges of involving people with HSU in co-design, such as recruitment and engagement, which is a finding consistent across co-design research [23, 36–38, 40–42]. Participants suggested better ways to support consumers, such as financial and practical support, kitchen table conversations, and parallel reference groups. Others have recommended careful preparatory planning of co-design, personal development support, flexible involvement, and relationship building that supports long term involvement could help ameliorate the problem of lack of consumer involvement [23, 36, 40, 41]. Participants desired diverse encounters that deepen their understanding of what it is like to experience HSU, and ultimately improve service design. Thus, co-design mechanisms and resources should support consumer involvement that is diverse, flexible, and deepens understanding [36, 38, 40, 41].

Diversity in co-design team membership is essential [43], however people who are hard to reach or from disadvantaged groups are often overlooked [37, 40]. Participants felt that diversity in co-design team membership was important as it enabled participants to listen and understand diverse perspectives and expertise. Participants reported that membership of this co-design team was diverse in terms of healthcare background yet identified an absence of people that are hard to reach or disadvantaged. Several participants described the team as comprising of Anglo-Saxon, professional, and two gendered (male and female). This runs counter to research which suggests diversity is essential if co-design outcomes are to be innovative [43], as different perspectives promote understanding and different ways of thinking [40, 44]. This finding suggests that diversity of professional backgrounds can take precedence over other types of diversity. We suggest those assembling co-design teams are purposive in their recruitment to ensure representation of diverse views, knowledge sources, and demographics, which is in line with findings which stress the importance of careful preparation of team membership [40, 43]. Following purposive recruitment, we recommend those reporting co-design outcomes should include descriptions of co-design team members to permit clear identification of diversity or lack thereof, as there is often inadequate reporting of co-design to enable this [17, 36, 44–46]. Additionally hard to reach or disadvantaged communities require co-design methodologies which align with their culture and values, such as co-design with First Nations Australians [47], people with severe mental health [48], or culturally and linguistically diverse communities [39]. Diverse co-design teams have been shown to overcome barriers to co-design involvement, such as discrimination, culture, complex multimorbidity and socioeconomic factors [36, 37, 40]. Perspectives of diverse participants are essential in service (re)design if health services are to be culturally safe and inclusive [36, 37, 39].

Relationships [42, 44, 49], learning [42, 44, 50], co-design purpose [8, 44], and managing expectations [23, 42, 44, 51] are essential for co-design success and these themes emerged in this study. Participants used words such as “trust” and “*understanding*” to describe relationships with co-design team members, which is similar with co-design research that found positive relationships help foster positive co-design environments [44, 49]. Conversely, “*professional jargon*” as described by one participant can impede understanding and participation [8]. Learning about “*different sides of the health system*” were described in interviews and has been reported in other co-design research as enabling co-design teams to deepen their understanding of different perspectives to move beyond existing boundaries [42, 44, 50]. Participants described a common purpose, yet some were unsure why they were invited to participate in this co-design or had role ambiguity. These finding contrast with co-design research which found managing expectations and role clarity were important for maintaining co-design engagement [8, 23, 44]. The slowness of co-design was raised by participants in interviews and has been highlighted in other co-design research as a barrier to co-design [42, 44, 50], yet essential for relationship and shared leadership development [44]. Some participants felt the COVID-19 pandemic impacted co-design momentum, their involvement and added an extra layer of complexity to an already complex environment. Most participants described previous (co-)design experience and subsequent expectations of what this co-design would involve. Since there are a plethora of co-design approaches with varying degrees of involvement [19, 20, 51], these findings suggest co-design organisers should describe co-design definition, principles, toolkits used and expectations to guide participants. Yet previous co-design research suggests co-design definitions and principles are often under-reported and overlooked [20, 21, 41].

This study has some limitations. There was a 50% response rate, therefore the views of non-participants may have been different. However, the backgrounds and experiences of all potential participants were represented in the sample population and all co-design governance structures were represented (Table 2). Because this was a small sample of known individuals, for ethical reasons we were unable to report characteristics of participants to whom quotes were attributed and we may not have been able to detect small differences in perceptions between subgroups, such as healthcare professionals compared to consumers. Participants who had a positive co-design experience may have been more likely to participate in this study. However, participants voiced a variety of views and experiences both positive and negative. Lastly, data collection occurred during the implementation phase of the Healthcare Connect North service, approximately 18-months into the co-design process. This could have led to some participant recall bias and participants may not recall their experiences correctly or their experience may have dulled over time.

## Conclusion

This study sought to explore the experiences and perspectives of people participating in the co-design of a community-based health service and contributes to the limited knowledge of the experiences and perspectives of co-design participants. While most participants reported positive aspects of their experience, they also perceived that there were factors which hindered the co-design process and others that could be improved, such as bureaucracy, greater diversity, and involvement of people with lived experience. Future co-design projects could use these findings to improve the co-design experience for participants, and ultimately the outcome for communities.

### Supplementary Information

**Supplementary Material 1.**

**Supplementary Material 2.**

## Data Availability

The datasets generated and/or analysed during the current study are not publicly available due to privacy and confidentiality but are available from the corresponding author on reasonable request.
