# Supplementary material for: Experiences of participants in the co-design of a community-based health service for people with high healthcare service use
Source: BMC Health Serv Res. 2024 Mar 14;24:339. doi: 10.1186/s12913-024-10788-5 (PMC10938828; doi:10.1186/s12913-024-10788-5)
Supplement: Supplementary file 2 — Supplementary Material 2. [file 12913_2024_10788_MOESM2_ESM.docx]

# **Evaluating the design process of a model for a community-based health service**

# **Interview Guide**

# **Introduction**

***Interviewer:*** Thanks for taking the time to meet with me today. We’re interested in your experiences and perspectives of participating in the design of Healthcare Connect North (formerly Complex Chronic Care). We’ve prepared some questions to facilitate our discussion, which I will be referring to throughout our discussion today.

Firstly, can I check if you have any questions about the study or interview process?

Can I confirm that you consent to participate today and have provided verbal consent or completed the Participant Consent Form?

***Interviewer*:** Before we start the interview, I’d like to collect a few details about you.

** If in person, give the Interviewee the Demographic Form to complete. If over the telephone or online, ask the demographic questions.*

| Which age range do you fall into? | | |
| --- | --- | --- |
| Please select one box: | | |
|  | | < 25 |
|  | | 25 – 34 |
|  | | 35 – 44 |
|  | | 45 - 54 |
|  | | 55 - 64 |
|  | | >= 65 years |
|  | | |
| Gender refers to current gender, which may be different to sex recorded at birth and may be different to what is indicated on legal documents. | | |
| Please select one box: | | |
|  | | Man or male |
|  | | Woman or female |
|  | | Non-binary |
|  | | [I/They] use a different term (please specify) ……………………………………………………………………………………………………………… |
|  | | Prefer not to answer |
|  | | |
| Are you of Aboriginal or Torres Strait Islander origin?  (For persons of both Aboriginal and Torres Strait Islander origin, mark both ‘Yes’ boxes). | | |
|  | No | |
|  | Yes, Aboriginal | |
|  | Yes, Torres Strait Islander | |
|  |  | |
| What category or categories best describe your background?  Please select one box: | | |
|  | | Healthcare consumer representative |
|  | | Allied health professional |
|  | | Policymaker |
|  | | Medical clinician |
|  | | Nursing clinician |
|  | | Other (please specify)  ……………………………………………………………………………………………………………………. |

***Interviewer:*** Thank you.

Are you happy for me to voice/video record our discussion for transcription purposes?

Thank you

****Audio/audiovisual recording begins***

# **Warm up question**

1. What’s your experience of managing chronic disease, either for yourself, your family or professionally?

# **Experience and expectations:**

1. Tell me about your experience of the design process?
2. Why do you think you were asked to take part in the design of this health service?

Additional question if needed:

- 1. What was your role?
  2. What made you decide to be involved?
  3. How do you think you contributed to the design process?
  4. What do you think were the goals of the design process?
     1. Do you think these were met? Why/why not?
  5. What's your understanding of the needs the model was trying to meet?
     1. Do you think the design to date as you understand it meets these needs?

1. What were your expectations of the project?

Additional question if needed:

- 1. To what extent where your expectations met?

# **Positive and challenging aspects:**

1. What were the positive things about the design process?

Additional question if needed:

- 1. What worked well?
  2. What aspects did you enjoy or find interesting or useful?

1. What aspects do you think didn’t work well?

Additional questions if needed:

- 1. Were the challenges? If so, what were they and what steps were taken to overcome these?

# **Team dynamics, conflict resolution, diversity, decision-making**

1. How diverse were the opinions and experiences voiced in the workshops, emails, and meetings?

Additional questions if needed:

- 1. What do you think about the diversity of people contributing?
  2. What do you think about the different experiences, characteristics, or backgrounds present (or not present)?
  3. What impact, if any, do you think diversity (or lack of) had on the process?

1. Did you feel you were able to contribute your opinion to discussions?
2. Did you think everyone was comfortable contributing? Why?

Additional questions if needed:

- 1. Did everyone get to speak?
  2. Was everyone heard?
  3. Was there any conflict?
  4. How was conflict resolved, if at all?

1. What were the group dynamics like?

Additional question if needed:

- 1. Did you know anyone else involved?
  2. How did you get to know each other?

1. How were the decisions made in the design process?

Additional question if needed:

- 1. How was consensus met?

1. Do you think everyone was engaged in the workshops, emails and meetings?

Additional question if needed:

1. Do you think this changed over time?
2. If not, what do you think prevented people from engaging?

# **Learning**

1. What did you learn from the design process?

Additional question if needed:

- 1. Have you changed the way you work, think or act since participating in the design process?

1. Would you get involved in a similar project again in the future?

# **Impact**

1. What do you believe the impact of the project is or will be?

Additional questions if needed:

- 1. Do you think it was it worthwhile? Why/why not?
  2. What’s your understanding of the outcomes from the design process?
  3. What do you think you contributed?
  4. What were the specific qualities you contributed to the discussion?

1. Do you feel you benefited from participating in the design process?

Additional question if needed:

- 1. Do you feel your participation impacted your team or the way you work?

# **Communication**

1. What was communication like?

Additional questions:

- 1. How did everyone communicate with each other?
  2. Did you receive adequate communication from the organisers?

# **Wrap up**

1. Is there anything else you wanted to add that we haven’t spoken about?

***Interviewer:*** Thank you for taking time out of your schedule to talk with me today. The audio/audiovisual recording will be transcribed, and I will check the transcription for accuracy. Would you like to receive a copy of the transcription to also check so you can amend or change any of your comments? If so, please return any comments within 14 days of receiving the transcript. If we don’t hear from you, we’ll assume you have no changes to make. If you have any other questions or concerns, please don’t hesitate to get in contact.

****Audio/audiovisual recording ends. Interview ends.***
